# Weight Status, Body Image and Bullying among Adolescents in the Seychelles

**DOI:** 10.3390/ijerph10051763

**Published:** 2013-05-02

**Authors:** Michael L. Wilson, Bharathi Viswanathan, Valentin Rousson, Pascal Bovet

**Affiliations:** 1 Centre for Injury Prevention and Community Safety (CIPCS), PeerCorps Trust Fund, 352/64 Makunganya Street, Co-Architecture Building 4th Floor, P.O. Box 22499, Dar es Salaam, Tanzania; E-Mail: michael.wilson@peercorpstrust.org; 2 Unit for Prevention and Control of Cardiovascular Disease, Ministry of Health, Victoria, Republic of Seychelles; E-Mail: vbharathyy@hotmail.com; 3 Institute of Social and Preventive Medicine (IUMSP), Lausanne University Hospital (CHUV), Lausanne, 1010, Switzerland; E-Mail: valentin.rousson@chuv.ch; 4 Institute of Social and Preventive Medicine (IUMSP), Lausanne University Hospital (CHUV), Lausanne, 1010, Switzerland

**Keywords:** overweight, obesity, bullying, sub Saharan Africa, epidemiology, school-health

## Abstract

We investigated the relationship between being bullied and measured body weight and perceived body weight among adolescents of a middle-income sub Saharan African country. Our data originated from the Global School-based Health Survey, which targets adolescents aged 13–15 years. Student weights and heights were measured before administrating the questionnaire which included questions about personal data, health behaviors and being bullied. Standard criteria were used to assess thinness, overweight and obesity. Among 1,006 participants who had complete data, 16.5% (95%CI 13.3–20.2) reported being bullied ≥3 days during the past 30 days; 13.4% were thin, 16.8% were overweight and 7.6% were obese. Categories of actual weight and of perceived weight correlated only moderately (Spearman correlation coefficient 0.37 for boys and 0.57 for girls; *p* < 0.001). In univariate analysis, both actual obesity (OR 1.76; *p* = 0.051) and perception of high weight (OR 1.63 for “slightly overweight”; OR 2.74 for “very overweight”, both *p* < 0.05) were associated with being bullied. In multivariate analysis, ORs for categories of perceived overweight were virtually unchanged while ORs for actual overweight and obesity were substantially attenuated, suggesting a substantial role of perceived weight in the association with being bullied. Actual underweight and perceived thinness also tended to be associated with being bullied, although not significantly. Our findings suggest that more research attention be given to disentangling the significant association between body image, overweight and bullying among adolescents. Further studies in diverse populations are warranted.

## Abbreviations

GSHS: Global School-based Health Survey
BMI: Body Mass Index
USD: United States Dollar
LMIC: Low- Middle-Income Country

## 1. Background

Overweight and bullying have risen to become important public health concerns among adolescent populations worldwide, as have the associated social and psychological issues [1]. Bullying, described as a repeated set of negative behaviors operating within an imbalance of power and directed with the intention of causing harm [2], can have severe and long term consequences for the health and well-being of those affected by it [3]. In a cross-national examination of bullying victimization in 40 countries, exposure to physical, verbal or indirect bullying ranged from 8.6% to 45.2% among boys, and from 4.8% to 35.8% among girls [4]. Adolescents who are overweight are at increased risk of developing various health complications such as diabetes and cardiovascular disease which also has effects which can last into adulthood [5]. The stages of adolescence are characterized by numerous hormonal and physiological transformations in the pathway from child to adult. During these processes, bodily appearances can become important components of individual self-esteem, psychological health and willingness to fully take part in social activities [6]. In western societies, the overwhelming prevalence of thin and lean female imagery and strong and lean male imagery in mass media have created widespread body image concerns [7]. In some low- and middle-income country countries (LMIC), particularly in the African region, body image ideals may have more complex social underpinnings [8]. Regardless of setting or culture, adolescents whose body types differ from socially prescribed norms sometimes fall victim to bullying behaviors from peers who tease or mock them in pointing out physical differences [9].

Recent research has shed light on the possible relationships between weight status, physical appearance and bullying victimization [10] with some authors suggesting that being victimized may also be associated with the image that one has of his or her own physical appearance [11,12]. Most available research on the association between body weight and bullying is primarily based mainly on data collected in high-income western settings. These studies have also tended to focus on females and rarely consider body type desirability as a potential modifier of risk relationships. In African settings, the relationship between body image and victimization is less clear [8]. Within the African diaspora, research has suggested that body image may be shaped to a greater extent by cultural preferences toward a larger body size, particularly for females. As a consequence, the harmful internalizing behaviors or stigma associated with a larger body size, may not be as pronounced in this population [8]. Both bullying behavior and overweight however, if left alone, can present important health risks to those affected, and perhaps to different extents in different populations. In the present study, we explored the association between actual weight, weight perception and being bullied during adolescence using a school-based sample from the Seychelles.

## 2. Methods

### 2.1. Population

The Republic of Seychelles constitutes a group of islands located approximately 1,800 km east of Kenya. The majority of the population is of black African descent, with minorities of European, Indian and Chinese origins. With a gross domestic product of $8,000 USD in 2007, the Seychelles is considered an upper middle-income country. Nationally, school attendance up to the 10th grade approaches 100%. Previous studies showed that the prevalence of the combined category of overweight and obesity increased from 10.2% to 16.1% between the years 1998 and 2004 [13,14].

### 2.2. The Global School-Based Health Survey

The data used in this study are derived from the 2007 Seychelles contribution to the Global School-based Health Survey (GSHS). The GSHS was developed by the World Health Organization in collaboration with the U.S. Centers for Disease Control. It collects relevant information for the discernment of behavioral and health correlates for adolescents of school age. The GSHS has been conducted in more than 40 countries and repeat surveys have been carried out to monitor changes in a number of them. The sampling framework of the GSHS is based on a two-stage sampling design with the first stage being a random selection of schools in the country and the second stage being a random selection of classrooms in each of the selected schools. The study is designed to enable each country to generate a representative sample of adolescents at the country level. The general methods of this survey have been previously published elsewhere [15]. In Seychelles, all 13 secondary schools including the age range of interest were included (in view of the small population), from which 64 classrooms were randomly selected (from a total of 274 classrooms), as described in previous papers on this same study in Seychelles [16,17]. All students in the selected classrooms were eligible to participate. In total, 1,432 students participated in the survey, corresponding to a participation rate of 82%. A self-administered anonymous questionnaire was given to each of the 1,432 participants (52% female), who ranged in age from 11–17 years. The research and ethical committee of the Ministry of Health had approved the study including the fact that parental informed consent was not necessary. The students were informed on the anonymous nature of the questionnaire and were free to participate.

### 2.3. Variables of Interest

The ascertainment of bullying victimization was derived from one question: “During the past 30 days, on how many days were you bullied?”. The responses were “0 day”; “1 or 2 days”; “3 to 5 days”; “6 to 9 days”; “10 to 19 days”; “20 to 29 days”; “All 30 days”. We dichotomized bullying into 0–2 days per month (77.7%) *vs.* three or more days per month (22.3%). Although arbitrary, this cut off takes into account the “repeated over time” nature of bullying [15]. This threshold has also been used in prior research [16] and is comparable with the existing literature on the subject [17].

The weights and heights of the students were measured by trained survey officers prior to administering the questionnaire using standard portable electronic scales and stadiometers. Values were written down on a piece of paper and given to the students before they completed their questionnaires. Students were requested to report their values on their individual anonymous answer sheets. Body weight categories in children were based upon BMI cut offs, which differ according to age and sex up to the age of 18 years. We used the BMI cut offs from the International Obesity Task Force for both overweight/obesity in children [18] and for thinness in children [19]. These cut off values in children and adolescents correspond to the BMI values in adults (starting at age of 18 years) of 18.5 (thinness), 25 (overweight) and 30 (obesity).

Perceived body image was derived from one closed-ended question reading: “How do you describe your weight: very underweight, slightly underweight, about the right weight, slightly overweight, or very overweight.” The categories of overweight and obesity were defined based on standardized sex and age specific body mass index (BMI) cut off values. Standard cut offs were used to assess thinness [18,20].

### 2.4. Analysis

We restricted the analyses to the 1,006 participants who had complete information for all the covariates of interest. Data were missing for 426 participants in total, including five for age, 15 for sex, 408 for measured weight and 55 for perceived weight. We calculated the distribution of categories of perceived weight and actual weight in relationship with being bullied. We then estimated the univariate and multivariate associations of actual weight and perceived weight with being bullied using logistic regression. To account for a possible clustering effect, a random classroom effect has been included using the command xtlogit in Stata 9.2 for all analyses (*i.e*., prevalence of bullying across categories of different variables and association between bullying in different variables). All analyses were performed using Stata 9.2 (StataCorp, College Station, TX, USA, 2011) for Windows XP^®^.

## 3. Results

Being bullied on at least 3 days during the 30-day recall period was reported by 16.5% (95%: confidence interval 13.3%–20.2%) of participants (males: 17.5% (12.2%–21.2%); females: 15.8% (12.4%–19.9%)). The proportions of students according to actual weight categories were as follows: thin 13.4% (10.9%–16.4%); overweight 15.6% (13.4%–17.9%); obese 7.7% (5.8%–10.0%).

**Figure 1:**
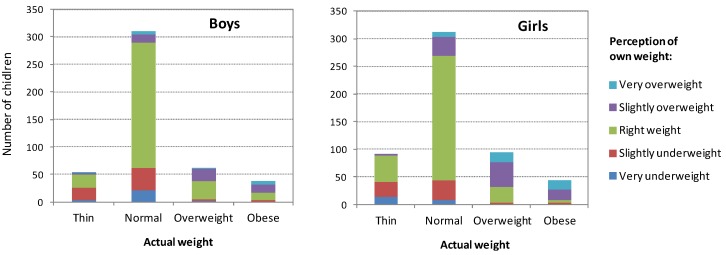
Relation between actual weight and perceived weight among male and female students aged 11–17 years.

Figure 1 shows that there were substantial proportions of students who perceived themselves as being larger, or thinner, than what their actual body weight indicated. This discrepancy between measured weight and perceived weight differed by sex. For example, among students with either normal weight, overweight and obesity, larger proportions of girls than boys perceived their weight as being too high. Inversely, a larger proportion of boys with normal weight perceived themselves as being underweight. Correspondingly, the Spearman coefficients of correlation between categories of actual weight (4 categories) *vs.* perceived weight (5 categories) were moderate: 0.38 in boys and 0.58 in girls (both *p* < 0.001).

Table 1 shows the proportions of students being bullied on at least 3 days in the 30 days preceding the survey according to categories of actual overweight and perceived overweight. There was a trend for bullying to be associated with both actual obesity status and perceived overweight. Confidence intervals are fairly large due to the limited number of participants in the overweight categories. The prevalence of bullying was also associated with younger age. The proportion of adolescents being bullied was almost identical in adolescents who had data missing, or not missing, in relation to the considered covariates.

**Table 1:** Prevalence (in percent) of male and female students aged 11–17 years being bullied on at least 3 days during the past 30 days, according to categories of actual weight, perceived weight, age and missing data status.

		N	Proportion (%)	95%CI
**Sex**				
	Boys	464	17.5	12.2–24.4
	Girls	542	15.8	12.4–20.0
**Actual body weight (BMI)**				
	Thin	145	11.8	4.7–26.7
	Normal	624	14.3	10.7–19.0
	Overweight	156	16.8	9.7–27.4
	Obese	81	31.2	7.8–41.1
**Perceived body weight**				
	Much too thin	50	16.5	3.4–52.7
	Slightly too thin	136	11.1	4.0–27.1
	About right	603	14.1	10.9–18.3
	Slightly too fat	158	18.1	10.3–29.9
	Much too fat	59	23.5	7.4–46.5
**Age**				
	11–12	165	18.1	10.6–29.5
	13–14	434	23.1	18.3–28.7
	15–17	407	11.3	8.6–14.8
**Missing data status**				
	Not missing	1006	16.5	13.3–20.2
	Missing	426	17.6	13.4–22.7

BMI: body mass index; Missing data on sex, age, actual BMI and perceived weight.

**Table 2:** Association between actual weight, perceived weight and being bullied among adolescents aged 11–17 years.

		Boys (n = 464)								Girls (n = 542)								Total (n = 1,006)						
		Model 1				Model 2				Model 1				Model 2				Model 1				Model 2		
		OR	95%CI	P		OR	95%CI	P		OR	95%CI	P		OR	95%CI	P		OR	95%CI	P		OR	95%CI	P
**Actual weight**																								
	Thin	1.20	0.55–2.62	ns		1.24	0.59–2.56	ns		1.26	0.67–2.38	ns		1.31	0.67–2.55	ns		1.27	0.77–2.07	ns		1.26	0.75–2.11	ns
	Normal	1	-	-		1	-	-		1	-	-		1	-	-		1	-	-		1	-	-
	Overweight	1.21	0.57–2.53	ns		0.91	0.43–1.91	ns		1.19	0.63–2.24	ns		0.95	0.46–1.97	ns		1.17	0.72–1.88	ns		0.90	0.53–1.53	ns
	Obese	1.52	0.63–3.69	ns		0.99	0.41–2.40	ns		2.09	1.00–4.41	0.051		1.50	0.68–3.59	ns		1.76	1.00–3.12	0.051		1.17	0.61–2.23	ns
**Perceived weight**																								
	Much too thin	1.93	0.68–5.46	ns		1.87	0.75–4.60	ns		1.12	0.36–3.50	ns		1.00	0.67–2.55	ns		1.50	0.71–3.16	ns		1.42	0.67–3.02	ns
	Slightly too thin	1.17	0.56–2.44	ns		1.21	0.60–2.41	ns		1.21	0.58–2.50	ns		0.95	0.46–1.97	ns		1.19	0.71–2.00	ns		1.12	0.66–1.90	ns
	About right	1	-	-		1	-	-		1	-	-		1	-	-		1	-	-		1	-	-
	Slightly too fat	1.95	0.93–4.06	ns		2.14	1.02–4.49	0.045		1.42	0.78–2.59	ns		1.38	0.68–2.78	ns		1.63	1.02–2.59	0.039		1.66	0.98–2.81	ns
	Much too fat	4.33	1.30–14.4	0.017		3.51	1.17–10.5	0.024		2.16	1.02–4.60	0.044		1.97	0.81–4.77	ns		2.74	1.45–5.18	0.002		2.71	1.35–5.42	0.005
**Age**																								
	11–12	1.86	0.72–4.81	ns		1.79	0.89–3.64	ns		2.59	1.32–5.07			2.46	1.24–4.87	0.010		2.05	1.15–3.64	0.014		2.07	1.16–3.68	0.013
	13–14	2.79	1.35–5.74	0.005		2.78	1.62–4.79	0.000		2.11	1.23–3.60			2.09	1.22–3.58	0.008		2.35	1.50–3.68	0.000		2.35	1.50–3.68	0.000
	15–17	1	-	-			-	-		1	-	-		1	-	-		1	-	-		1	-	-

%: proportion in percent; OR: odds ratio; 95%CI: 95% confidence interval. Model 1: univariate ORs for models including either actual weight, perceived weight or age; Model 2: multivariate ORs for models including all variables in the table, including sex for model with all participants. Being bullied is defined as being bullied on at least 3 days in the past 30 days.

Table 2 shows the univariate (Model 1) and multivariate (Model 2) relationships of being bullied with actual weight and perceived weight. In univariate analysis (Models 1 in Table 2), there was a trend toward a J-shaped association between being bullied and both perceived weight and actual weight, both in boys and girls. The ORs were generally larger for perceived weight than for actual weight. The associations were statistically significant for perceived weight among both boys and girls and nearly statistically significant for actual obesity among girls and all participants. Bullying was also associated with younger age in both boys and girls. Of note, the numbers of participants in the high and low categories for actual weight and perceived weight are small, which limits the power of univariate analyses in these categories.

When adjusting for both actual weight and perceived weight (Models 2 in Table 2), and as well as for age, the ORs for actual weight were substantially attenuated as compared to the ORs found in the univariate analysis, particularly among boys, with none of the estimates reaching statistical significance. In contrast, the ORs for perceived overweight were virtually unchanged and associations were statistically significant for boys and for all participants. In other words, variation in weight perception at any fixed level of actual body weight was a predictor of being bullied, while variation in actual weight at any fixed level of perceived weight did not predict being bullied (at least among boys). These findings suggest that perceived weight is more strongly associated with being bullied than actual weight. While not a focus of the analyses, age was also significantly associated with bullying independent of actual or perceived weight.

## 4. Discussion

The prevalence of being bullied among adolescents seemed to be higher in Seychelles than in some European countries [4], but lower than in some other countries in the African region [21]. Our findings suggest that being bullied is associated more strongly with the perception of being overweight compared to actual overweight status. These findings are the first, to our knowledge, to examine the relationship between being bullied, actual weight and perceived weight among adolescents in the African region. These results can have implications for policy and programs to prevent bullying among adolescents.

The fairly low prevalence of being bullied in Seychelles, compared to other countries in the African region [21], may be related to a number of diverse factors such as the small size of the country, with active involvement of most people (and adolescents) in small communities, a socially tolerant Creole culture, and long standing social and political stability in the country [17]. The higher prevalence of bullying among boys compared with girls is consistent with other studies [4]. Prior research in Seychelles using a lower threshold for bullying (one or more days in a 30 month period) already suggested these results [22]. We also found that bullying was more frequent among younger adolescents in line with findings from other research suggesting that bullying behavior was most frequent between the ages of 11–13 years as opposed to later adolescence [23]. This may be due to younger children having more student fellows who are older than them in school and in a position to bully them [24].

We observed that being bullied was associated with both measured obesity and perceived overweight. The association tended to be stronger with perceived weight than with actual overweight, particularly among boys. This contrasts with prior research suggesting that bullying behavior may have conferred greater social status to larger boys [25,26]. Previous reporting in Seychelles showed that male adolescents tended to have larger body size ideals than did girls, which boys may associate with strength and attractiveness [27]. The degree to which a large body type ideal is related with robust stature and physical musculature as opposed to overweight has not yet been studied in Seychelles. However, other research suggests that body build rather than overweight may be desirable among boys [28].

The association between perceived body weight and bullying victimization is consistent with the relationship between body perception and peer group values and social influences. Peer group pressure, which is an important socializing force during adolescence [29], can profoundly shape the self-perception of one’s body weight [28]. Adolescents seeking to be in desirable peer groups may be confronted with concerns about their body sizes, which may result in insecurity, visible anxiety and low self-esteem, each of which is associated with higher rates of victimization [30]. The social influences of the cultural context serve to reinforce certain peer group values towards preferring a certain body size ideal [31].

Preference for a thin body size in Seychelles is consistent with prior research in Seychelles [20], and in line with findings in high-income Western settings [32]. Preference for a thin body size, at least among females, may however be atypical of other countries in the African region, and even of other populations of the African diaspora, in which a large body size seems to be valued for women [33,34]. Cultural norms favoring a large body size, can be an important factor for sustaining overweight in the population and can have complex implications. For example, a large body size can serve, in some cultural contexts, to legitimize a consenting eligible female as being ready for married life [35,36]. The values attached to larger body size ideals may include increased social status (for themselves and their families) related to the perception of greater wealth and better health [37].

We also found that both actual low body weight and perceived low body weight tended to be associated with being bullied, although the relationship was not statistically significant in our data, consistent with previous research [26].

## 5. Strengths and Limitations

This study has several strengths. The data on perception were collected using a standardized questionnaire that has been validated cross-nationally, actual body weight was measured (*i.e*., not reported), and the sample size was representative of the general population of adolescents in the Seychelles. To our knowledge, no previous study among adolescents in the African region has examined the relationship between body image perception, actual weight and being a victim of bullying. There are also several limitations. There was a substantial amount of missing data, mainly related to our reliance on requesting participants to reporting their measurements of body weight and height performed before the interview. A main limitation is that only few participants fell into the highest or lowest categories of actual weight and/or perceived weight, limiting the power of statistical analyses. Larger studies will be needed to further explore this question. Another limitation is that bullying and perceived weight were based each on a sole question. Further studies should assess the associations between bullying and perceived and actual weight in more detail. The cross-sectional nature of the data warrants caution when disentangling the respective roles of actual weight *vs.* perceived weight. More generally, actual and/or perceived weight can be both a cause and a consequence of being bullied. While both actual overweight or perceiving oneself as overweight are likely causes of being bullied among adolescents, becoming overweight (as a coping or defensive mechanism) or perceiving oneself as overweight may also be consequences of being bullied. Longitudinal studies with more comprehensive data to characterize weight perception will be needed to further disentangle these factors.

However, these concerns do not alter our conclusions that both actual weight and perceived weight are associated with being bullied (whether as a cause or as a marker) and that these factors should be addressed when designing interventions to reduce victimization on the basis of body weight or negative self-image. Areas for intervention may include weight control through dietary and physical activity measures, addressing the perception of one’s weight through adequate psychosocial and/or cultural interventions with the goal that adolescents can better cope with their weight. Whether being bullied results as a consequence of being overweight, or whether one intentionally gains weight to avoid being victimized, are questions which have yet to be answered by the literature [38]. Finally, an improved understanding of body weight perceptions in communities with a dual burden of overweight and bullying is useful if programs for either are to be successful.

## 6. Conclusions

This study provides information on the contributions of actual body weight and body size perception to bullying victimization. The results, while informative, are likely to be most useful in their ability to generate further hypotheses to better understand the association between bullying and body image among adolescents in different contexts. For example, further research should examine the context and age at which these factors begin to contribute to the overall bullying risk profile. Potentially, studies that examine body image perceptions given peer, family, neighborhood, regional and societal influences, might be useful. Finally, further research on the link between actual and perceived body size and being bullied is warranted in other populations as well as over time in same populations in view of changes in social and cultural norms between populations and over time.
